# Impact of *CYP2C19* Variants on Clinical Efficacy of Clopidogrel and 1-Year Clinical Outcomes in Coronary Heart Patients Undergoing Percutaneous Coronary Intervention

**DOI:** 10.3389/fphar.2016.00453

**Published:** 2016-11-24

**Authors:** Hong Sun, Qiang Qu, Zhen-Fan Chen, Sheng-Lan Tan, Hai-Jun Zhou, Jian Qu, Hui Chen

**Affiliations:** ^1^Department of Pharmacy, The Second Xiangya Hospital, Central South UniversityChangsha, China; ^2^Institute of Clinical Pharmacy, Central South UniversityChangsha, China; ^3^Department of Pharmacy, Provincial Clinical College of Fujian Medical University, Fujian Provincial HospitalFuzhou, China; ^4^Department of Pharmacy, Xiangya Hospital, Central South UniversityChangsha, China; ^5^Hypertension Laboratory, Provincial Clinical College of Fujian Medical University, Fujian Provincial Cardiovascular Disease Institute, Fujian Provincial HospitalFuzhou, China

**Keywords:** CYP2C19, clopidogrel, PCI, antiplatelet therapy, metabolizers

## Abstract

The impact of pharmacogenetic variants of cytochrome P450 2C19 (*CYP2C19*) on clopidogrel-mediated effects on platelet inhibition, inflammatory response and endothelial function, as well as risk of major adverse cardiovascular events (MACE), in coronary heart patients undergoing percutaneous coronary intervention (PCI) was investigated. To this end, we assessed the residual platelet aggregation rate (RPA), maximal aggregation rate (MAR) and plasma levels of sCD40L, sP-selectin, MMP-9, sVCAM-1 and sE-selectin after 24 h of PCI in 559 patients treated with clopidogrel and followed up for 1 year for evidence of MACE. *CYP2C19* ^*^2 and ^*^3 variants were identified using a clopidogrel-sensitive gene detection kit. Our results showed higher RPA and MAR as well as increased sE-selectin, sCD40L, sP-selectin, MMP-9, and sVCAM-1 levels in CYP2C19 intermediate metabolizer (IM, CYP2C19^*^1/^*^2, or ^*^1/^*^3), poor metabolizer (PM, CYP2C19^*^2/^*^2, ^*^2/^*^3, or ^*^3/^*^3) and combined IM+PM groups, relative to those in extensive metabolizers (EM, CYP2C19^*^1/^*^1). In total, 519 patients completed 1 year of follow-up, among which 69 (13.3%) experienced MACE. The risk of MACE in CYP2C19 IM+PM patients was 2.664 times higher than that in CYP2C19 EM patients (OR = 2.664 (1.397–5.193), *P* = 0.004). The data suggest that CYP2C19^*^2 and ^*^3 variants modulate the drug efficacy of clopidogrel in coronary heart patients undergoing PCI and further enhance the risk of MACE. Accordingly, *CYP2C19* pharmacogenetic profiling may be beneficial for coronary heart patients undergoing PCI to predict the efficacy of treatment with clopidogrel. We propose that IM and PM patients should benefit from treatment with higher clopidogrel doses to improve efficacy and reduce the incidence of MACE.

## Introduction

Owing to dietary and lifestyle changes, the number of coronary heart disease (CHD) patients worldwide has increased over the years. Percutaneous coronary intervention (PCI) is one of the most significant developments in the treatment of CHD. However, stent thrombosis (ST) is a major complication affecting the efficacy of PCI. Currently, anti-platelet drugs, such as clopidogrel and aspirin, are used to prevent the occurrence of ST after PCI (Stone et al., 2013). Post-operative joint use of clopidogrel and aspirin antiplatelet therapy has been shown to reduce ST, death, myocardial infarction and other major adverse cardiovascular events (MACE), and has rapidly become standard treatment (Steinhubl et al., 2002; Han et al., 2015). However, a number of patients taking conventional doses of clopidogrel continue to experience severe coronary ischemic events (Müller et al., 2003; Stone et al., 2013). The rate of clopidogrel resistance is reported to range from 4.2 to 27.8% (Järemo et al., 2002; Serebruany et al., 2005), implying individual differences in the antiplatelet efficacy of clopidogrel.

Several factors affect the antiplatelet activity of clopidogrel, including inter-patient compliance, age, diabetes, smoking, and drug interactions (Sabaté et al., 2015). Although the efficacy of clopidogrel is linked to several enzymes, transporters and acceptors, such as CYP2B6, CYP3A4, CYP3A5, ABCB1, PON1, and P2Y12, the most important factors suggested to be associated with low response to the drug are CYP2C19^*^2 and CYP2C19^*^3 variants (Nagashima et al., 2013; Jiang et al., 2015; Sabaté et al., 2015; Ma et al., 2016). The impact of *CYP2C19* variants on risk of MACE is controversial (Mega et al., 2009, 2010b; Bauer et al., 2011; Han et al., 2015). A systematic review and meta-analysis by Bauer et al. (2011) revealed no substantial or consistent influence of *CYP2C19* variants on the clinical efficacy of clopidogrel and risk of MACE. In contrast, the groups of Mega and Han reported significantly increased risk of MACE in association with CYP2C19^*^2 and ^*^3 variants (Mega et al., 2010b; Han et al., 2015; Khalil et al., 2016).

Clopidogrel, an irreversible inhibitor of the adenosine diphosphate (ADP) platelet receptor, P2Y12, is widely used to prevent thromboembolic events in patients (Wei et al., 2015). The platelet function test is usually employed to detect the antiplatelet efficacy of clopidogrel. ADP-induced optical turbidity has been the “gold standard” to evaluate the efficacy of clopidogrel for several decades (Steinhubl et al., 2002). Clopidogrel is reported to block the platelet ADP receptor to inhibit platelet aggregation, which reduces the expression and release of plasma-soluble CD40 ligand (sCD40L) and P-selectin, from platelets, preventing further platelet activation (Li et al., 2015; Obradovic et al., 2015). SCD40L is mainly generated by cleavage of CD40L from the surface of activated platelets, and is therefore considered a platelet activation marker (Gremmel et al., 2015; Li et al., 2015). P selectin is mainly located in α-particles of platelets and stick tubular bodies (Weibel-Palade) of endothelial cells (Kaufmann et al., 2013). During platelet activation and endothelial damage, the protein is rapidly expressed on the cell surface, thereby mediating leukocyte, platelet, and endothelial cell adhesion (Kaufmann et al., 2013).

The mechanism underlying ST after PCI is complex. Anti-platelet drugs only act on a specific part of this complex network (Antonino et al., 2009). Chronic inflammation and endothelial damage are important steps in the pathogenesis of thrombosis after PCI (Antonino et al., 2009; Willoughby et al., 2014). Clopidogrel exerts not only anti-inflammatory activity but also protective effects on the vascular endothelium (Liu et al., 2012; Willoughby et al., 2014). The drug protects the endothelium by hindering the expression of vascular cell adhesion molecule-1 (VCAM-1) that is induced by a variety of cytokines (Yang et al., 2016). E-selectin, a cell adhesion molecule located on the vascular endothelial cell surface, is the first to be expressed in the inflammatory reaction, in turn causing aggravation of endothelial damage, enhanced inflammation, plaque rupture and thrombosis (Smadja et al., 2012). Increased levels of the adhesion molecule, gelatinase matrix metalloproteinase-9 (MMP-9), an inflammatory marker, can induce plaque rupture, resulting in thrombosis (Sternberg et al., 2016).

Considering this background, we aimed to investigate whether *CYP2C19* variants are involved in (1) clopidogrel-mediated platelet effects, (2) clopidogrel-mediated inflammatory endothelial effects, and (3) risk of MACE.

## Methods

### Patients and study design

Patients were enrolled from Fujian Provincial Hospital between March 2012 and March 2014. The ethics committee of the hospital approved the study and all patients provided written informed consent. Procedures were carried out in accordance with the approved guidelines. This prospective study was designed to determine the effects of *CYP2C19* variants on platelet reactivity, inflammatory and endothelial biomarkers and 1-year clinical outcomes in CHD patients undergoing PCI. Patients were eligible for study enrollment in the cases where (1) ages were higher than 18 and (2) they were diagnosed with CHD using coronary angiography and successfully treated with PCI. Exclusion criteria were as follows: (1) aspirin, clopidogrel and contrast agent allergy, (2) severe diseases, such as tumors and bleeding disorders, (3) hemodynamic instability (systolic blood pressure <90 mmHg and/or diastolic blood pressure <50 mmHg), severe ventricular dysfunction (LVEF <40%), (4) severe liver and kidney dysfunction, (5) blood disease or bleeding diathesis, platelet count <100 × 10^9^/L and hematocrit <30%.

### Platelet function test

After 24 h of PCI, 3 ml peripheral blood samples were collected and mixed with 3.8% trisodium citrate, and platelet aggregation was determined via the turbidimetric method in 2 h with a four-channel light transmission aggregometer (LBY-BJ4; Precil, Beijing, China). Platelet aggregation was stimulated with 5 μmol/L adenosinediphosphate (ADP). After agonist stimulation, the degree of light transmission was monitored. Results were recorded as light transmission at maximal aggregation. The platelet aggregation rate was recorded as a curve for 5 min and expressed as residual platelet aggregation rate (RPA).

After 24 h of PCI, creatinine (Cr), lipids (TC, TG, HDL-c, LDL-c), platelet count (PLT), RPA, and fibrinogen (FIB) levels were tested in the hospital laboratory and plasma levels of sCD40L, sP-selectin, MMP-9, sVCAM-1, and sE-selectin were assessed via enzyme-linked immunosorbent assay (ELISA) (HUAMEI, Wuhan, China) according to the manufacturer's instructions.

### PCI and clopidogrel treatment

PCI was performed in accordance with the standard of care. All patients were administered 300 mg clopidogrel and 40 mg atorvastatin prior to the operation. The operator decided on the choice of anticoagulant and the use of glycoprotein IIb/IIIa inhibitor (tirofiban), as well as the type of stent. After PCI, patients were treated with 100 mg/day aspirin indefinitely and 75 mg/day clopidogrel for 1 year. We defined the success of PCI as follows: (1) achievement of complete revascularization and residual stenosis of <20% in target vessels of patients, (2) level 3 TIMI blood flow, (3) partial or complete remission of symptoms of myocardial ischemia, and (4) no serious complications during hospitalization (such as acute myocardial infarction, urgent target lesion revascularization or death).

### Genotype analysis

*CYP2C19* genotypes were identified using the clopidogrel-sensitive gene detection kit (BAIO company, Shanghai, China). DNA was extracted from whole peripheral blood, followed by stepwise evaluation involving single-base primer extension, PCR amplification chip spotting, mass spectrometry, DNA microarray and analysis of data with software to obtain *CYP2C19* genotypes. All steps were performed in strict accordance with the BAIO genetic analyzer manual instructions. According to the CYP2C19^*^1, ^*^2, ^*^3 genotypes, patients carrying *1/^*^1 were grouped as extensive metabolizers (EM), ^*^1/^*^2 or ^*^1/^*^3 as intermediate metabolizers (IM), and ^*^2/^*^2, ^*^2/^*^3 or ^*^3/^*^3 as poor metabolizers (PM).

### Clinical endpoints

The clinical endpoints include the following MACE: cardiac death, myocardial infarction, and unstable angina. Cardiac death was defined as any death due to cardiovascular causes or not clearly attributable to non-cardiovascular causes. Myocardial infarction was defined as recent ischemic symptoms with ST-segment elevation or depression and abnormally elevated troponin. Unstable angina was defined as resting onset, duration greater than or equal to 20 min or more severe and longer duration of angina than before and higher frequency of onset. Diagnosis was performed during hospitalization according to serum creatine kinase levels, electrocardiogram findings and other indicators.

### Statistical analysis

Statistical analysis was carried out using SPSS software (version 11.0 for Windows; SPSS, Chicago, IL). Continuous data, presented as median and interquartile range, were compared using Kruskal-Wallis one-way analysis of variance or Mann-Whitney U test. Measurement data following normal distribution were expressed as means ± standard deviation (x ± s) and compared using *t*-test or univariate analysis of variance (ANOVA). Categorical variables were compared with the chi-square or Fisher exact test, as appropriate. Time-to-event outcomes were analyzed via Kaplan-Meier analysis and differences compared with the log-rank test. Logistic regression analysis was performed to assess independent predictors of MACE. *P* < 0.05 were considered statistically significant.

## Results

### Study population

**Figure 1:**
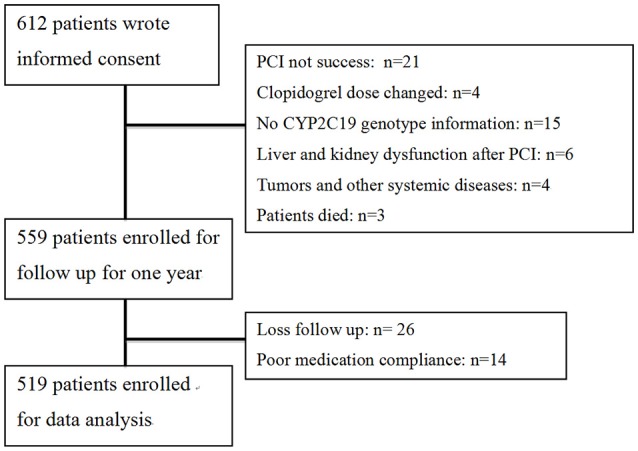
**Flow diagram of the study**.

A flow diagram of the study is shown in Figure 1. A total of 612 patients provided written informed consent. Based on inclusion criteria, 559 of these patients were enrolled for 1-year follow-up. Owing to loss to follow-up and poor medication compliance, 40 patients were further excluded, leading to a final total of 519 study participants. The follow-up rate was 95.3%. Clinical characteristics of patients are presented in Table 1. According to CYP2C19 genotype, 181, 253, and 85 patients were grouped as EM, IM, and PM, respectively, for further study. Comparison of clinical characteristics among the three groups disclosed no significant differences in age, gender, body mass index (BMI), CHD risk factors (hypertension, diabetes, smoking) and previous history of myocardial infarction (*P* > 0.05). Levels of lipids, creatinine, platelet count and fibrinogen were comparable among the three groups (*P* > 0.05). Differences in usage of clinical medicines, including ACEI/ARB, β-blockers, CCB, statins, PPI, and tirofiban (*P* > 0.05) were not statistically significant. Moreover, left main coronary artery lesions, vascular lesions (≧3), frame lengths and bracket numbers were similar among the three groups (*P* > 0.05).

**Table 1:** **Clinical characteristics of clopidogrel-treated patients with various genotypes of CYP2C19 after PCI**.

**Parameters**	**Patient numbers (*N* = 519)**	**CYP2C19 genotypes**			***P*-value**
		**EM (*N* = 181)**	**IM (*N* = 253)**	**PM (*N* = 85)**	
Age (years)	64.6±10.8	64.9±10.8	64.0±10.8	65.7±10.9	0.421
Male (%)	404 (77.8)	139 (76.8)	199 (78.7)	66 (77.6)	0.899
Body mass index (kg/m^2^)	23.91±2.80	23.74±2.90	24.11±2.77	23.66±2.89	0.267
Hypertension	334 (64.4)	118(65.2)	165 (65.2)	51 (60.0)	0.657
Diabetes	182 (35.1)	63 (34.8)	89 (35.2)	30 (35.3)	0.996
Smoking	240 (46.2)	77 (42.5)	121 (47.8)	42 (49.4)	0.450
Old myocardial infarction	103 (19.8)	42 (23.2)	42 (16.6)	19 (22.4)	0.193
CHOL (mmol/l)	4.27±1.33	4.19±1.06	4.38±1.54	4.15±1.15	0.218
TG (mmol/l)	1.70±1.16	1.63±1.01	1.75±1.30	1.69±1.00	0.576
HDL (mmol/l)	1.05±0.27	1.06±0.27	1.04±0.27	1.04±0.27	0.772
LDL (mmol/l)	2.72±0.92	2.68±0.95	2.77±0.86	2.65±1.04	0.456
CR (umol/l)	81.1±23.0	80.9±20.8	81.8±25.4	79.2±19.5	0.649
PLT (× 10^9^)	217.8±60.6	214.1±61.1	211.3±58.4	216.3±65.9	0.456
Fg (g/l)	3.56±1.02	3.53±1.10	3.57±0.97	3.58±1.01	0.911
**THERAPY AT DISCHARGE (%)**					
ACEI/ARB	425 (81.9)	147 (81.2)	213 (84.2)	65 (76.5)	0.267
Beta-blocker	447 (86.1)	156 (86.2)	221 (87.4)	70 (82.4)	0.514
CCB	119 (22.9)	42 (23.2)	54 (21.3)	23 (27.1)	0.552
Statins	519 (100)	181 (100)	253 (100)	85 (100)	
PPI	519 (100)	181 (100)	253 (100)	85 (100)	
Tirofiban	116 (22.4)	36 (19.9)	54 (21.3)	26 (30.6)	0.129
Naoxintong	171 (32.9)	58 (32.0)	80 (31.6)	33 (38.8)	0.450
**PCI (%)**					
Left main coronary artery lesion	28 (5.4)	10 (5.5)	13 (5.1)	5 (5.9)	0.962
Vascular lesion ≥ 3	260 (50.1)	82 (45.3)	132 (52.2)	46 (54.1)	0.266
Bracket number	1 (1)	1 (1)	1 (1)	2 (1)	0.081
Frame length (mm)	33.0 (29.0)	33.0 (28.0)	38 (29.5)	38 (36.5)	0.064

*Data are expressed as means ± SD or numbers (%). TC, total cholesterol; LDL, low-density lipoprotein; HDL, high-density lipoprotein; ACEI, angiotensin-converting enzyme inhibitor; ARB, angiotension II receptor blocker; CCB, calcium channel blockers; PPI, proton pump inhibitor; CR, serum creatinine; PLT, platelet count; Fg, fibrinogen; EM, extensive metabolizers; IM, intermediate metabolizers; PM, poor metabolizers*.

### Effects of CYP2C19 metabolizers on the drug efficacy of clopidogrel

**Figure 2:**
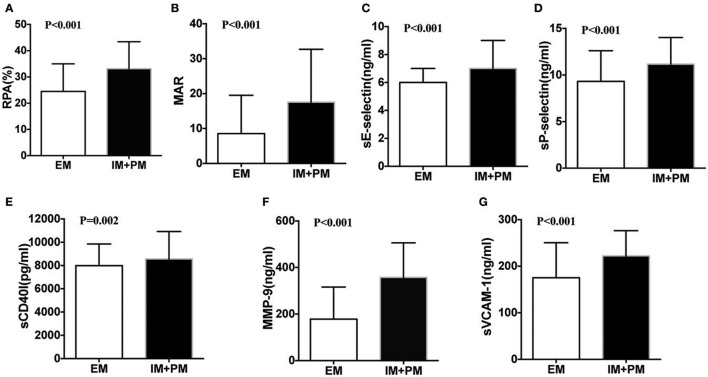
**Comparison of residual platelet aggregation indexes in patients stratified by CYP2C19 metabolizers after PCI. (A)** RPA, **(B)** MAR, **(C)** sE-selectin, **(D)** sP-selectin, **(E)** sCD40l, **(F)** MMP-9, and **(G)** sVCAM-1 between EM and IM+PM patients.

After 24 h of PCI, we measured levels of RPA, MAR, sE-selectin, sCD40L, sP-selectin, MMP-9, and sVCAM-1 in all patients. Significant differences were observed among the three groups using Kruskal-Wallis one-way analysis of variance (Table 2). All the parameters examined showed a higher trend in PM than IM patients. Comparison of these parameters in the EM and IM+PM groups revealed significant differences (Table 2, Figure 2), with higher levels in IM+PM than in EM patients (All *P* < 0.05).

**Table 2:** **Comparison of residual platelet reactivity, inflammatory, and endothelial biomarkers in clopidogrel-treated patients from various CYP2C19 metabolizer groups after PCI**.

**Parameters**	**CYP2C19 metabolizers**				*P*-value^*^	***P*-value^#^**
	**EM (*N* = 181)**	**IM (*N* = 253)**	**PM (*N* = 85)**	**IM+PM (*N* = 338)**		
RPA	8.58 (17.19)	15.89 (27.68)	23.39 (30.84)	17.53 (28.55)	*P* < 0.001	*P* < 0.001
MAR	24.49 (24.82)	33.04 (29.54)	33.66 (38.11)	33.13 (30.48)	*P* = 0.001	*P* < 0.001
sE-selectin (ng/ml)	5.98 (1.84)	7 (2)	7 (3)	7 (3)	*P* < 0.001	*P* < 0.001
sCD40L (pg/ml)	7989.1 (4397.4)	8819.5 (4488.0)	8440.0 (5906.0)	8560.95 (4681.52)	*P* = 0.001	*P* = 0.002
sP-selectin (ng/ml)	9.31 (5.99)	11.03 (6.08)	11.89 (6.89)	11.67 (6.12)	*P* = 0.035	*P* < 0.001
MMP-9 (ng/ml)	178.11 (223.89)	361.34 (143.54)	348.38 (224.18)	357.22 (228.89)	*P* < 0.001	*P* < 0.001
sVCAM-1 (ng/ml)	175.25 (133.16)	216.63 (106.77)	233.91 (112.19)	222.06 (111.29)	*P* < 0.001	*P* < 0.001

Values are median (interquartile range). RPA, residual platelet aggregation rate; MAR, maximum platelet aggregation rate; EM, extensive metabolizers; IM, intermediate metabolizers; PM, poor metabolizers. *P*-value^*^, Kruskal-Wallis one-way analysis comparing EM, IM and PM; P-value^#^, Mann-Whitney U-test comparing EM and IM+PM.

### Effects of CYP2C19 metabolizers on clinical outcomes

**Figure 3:**
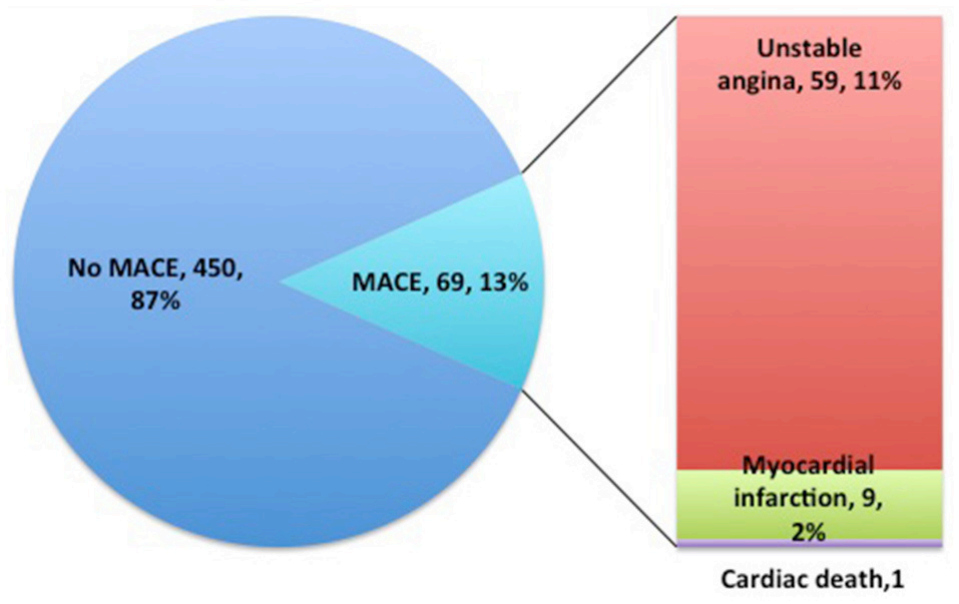
**One-year follow-up clinical outcome distributions of clopidogrel-treated patients after PCI**.

**Figure 4:**
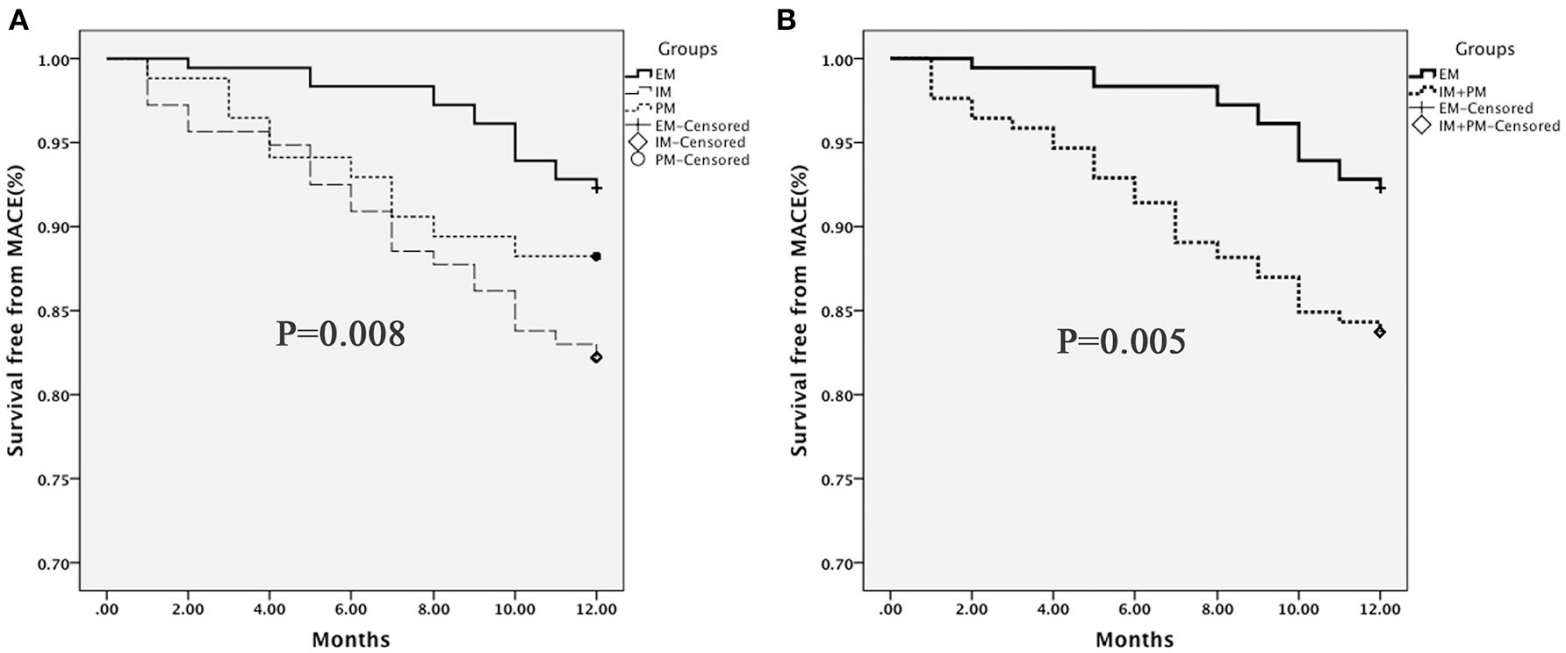
**MACE-free survival in patients stratified by CYP2C19 metabolizer status. (A)** EM vs. IM vs. PM; **(B)** EM vs. IM+PM. Differences were compared using the log-rank test.

One-year follow-up was completed in 559 patients. The total clinical outcomes are shown in Figure 3. During the follow-up period, 69 patients (13.3%) experienced adverse cardiovascular events (1 cardiac death, 9 myocardial infarction and 59 unstable angina). The total frequency of MACE was different among EM, IM and PM patient groups (7.8% vs. 17.8% vs. 11.8%, *P* = 0.009). The frequency of unstable angina differed among EM, IM and PM patients (6.6% vs. 15.4% vs. 9.8%, *P* = 0.014). We observed a strong trend toward higher frequency of unstable angina and MACE in IM patients, compared to PM patients. Comparison of the total frequencies of MACE and unstable angina in EM and IM+PM groups additionally revealed significant differences (Table 3). MACE-free survival was further analyzed in patients groups stratified by *CYP2C19* metabolizer status. As shown in Figure 4, CYP2C19 EM patients exhibited significantly lower rates of MACE, compared with patients in the IM, PM, and combined IM+PM groups.

**Table 3:** **Comparisons of MACE among different CYP2C19 metabolizer patient groups**.

**Parameters**	**CYP2C19 metabolizers**				*P*-value^*^	***P*-value^#^**	**OR (Odds ratio (95%CI))^#^**
	**EM (*N* = 181)**	**IM (*N* = 253)**	**PM (*N* = 85)**	**IM+PM (338)**			
Cardiac death	1 (0.6%)	0	0	0	0.392	0.354	–
Myocardial infarction	1 (0.6%)	6 (2.4%)	2 (2.4%)	8 (2.4%)	0.32	0.172	1.374 (1.081–1.746)
Unstable angina	12 (6.6%)	39 (15.4%)	8 (9.4%)	47 (13.9%)	0.014	0.013	1.259 (1.088–1.458)
Total	14 (7.8%)	45 (17.8%)	10 (11.8%)	55 (16.3%)	0.009	0.006	1.267 (1.1.3–1.456)

Abbreviations are the same as for Table 2. P-value^*^, Chi-square or Fisher exact test comparing EM, IM, and PM; P-value^#^, Chi-square or Fisher exact test comparing EM and IM+PM.

### Univariate and multivariate logistic regression analysis of the impact factors of MACE after PCI

Using MACE after PCI as the dependent variable and age, BMI, CHD risk factors (hypertension, diabetes, smoking), vascular lesions ≥ 3, frame length, bracket number, levels of platelet activity indicators after PCI and CYP2C19 metabolizers (IM+PM and EM) as independent variables, we analyzed whether these relevant parameters influence the occurrence of MACE. Univariate logistic regression analysis showed significant association of CYP2C19 metabolizers with occurrence of MACE (OR = 2.318 (1.251–4.297), *P* = 0.008) (Table 4). Multivariate logistic regression analysis further disclosed that smoking, frame length and CYP2C19 IM+PM genotypes are significantly associated with risk of MACE (*P* = 0.008, *P* = 0.028, *P* = 0.004, respectively). Notably, the risk of MACE in CYP2C19 IM+PM patients was 2.664 times higher than that in CYP2C19 EM patients (OR = 2.664 (1.397–5.193), *P* = 0.004) (Table 5).

**Table 4:** **Univariate analysis of variables in relation to occurrence of MACE after PCI**.

**Variables**	**OR**	**(95%CI)**	***P*-value**
Age	1.073	0.645–1.785	0.787
BMI	1.011	0.924–1.106	0.825
Hypertension	0.785	0.467–1.317	0.359
DM	0.915	0.535–1.565	0.746
Smoking	1.006	0.605–1.673	0.981
Vascular lesion ≥ 3	1.347	0.808–2.245	0.253
Frame length (mm)	1.009	0.998–1.020	0.108
Bracket number	1.249	0.908–1.719	0.172
RPA	0.992	0.975–1.009	0.337
sCD40L	1	1.000–1.000	0.638
sP-selectin	0.991	0.956–1.028	0.633
MAR	1.001	0.987–1.015	0.911
MMP-9	1.001	1.000–1.001	0.05
sVCAM-1	1.001	1.001–1.003	0.116
sE-selectin	1.026	0.960–1.096	0.446
CYP2C19 metabolizers	2.318	1.251–4.297	0.008

*Abbreviations as for Tables 1, 2*.

**Table 5:** **Multivariate analyses of variables in relation to occurrence of MACE after PCI**.

**Variables**	**OR (95% CI)**	***P*-value**
The number of coronary lesions	0.015 (0.687–2.172)	0.003
Smoking	0.958 (0.928–0.989)	0.008
Frame length (mm)	1.030 (1.003–1.058)	0.028
Bracket number	1.039 (0.575–1.878)	0.898
CYP2C19 metabolizers	2.664 (1.397–5.193)	0.004

*Abbreviations as for Tables 1, 2*.

## Discussion

The current study focused on determining whether *CYP2C19* genetic variants modulate clopidogrel-mediated antiplatelet effects and risk of MACE in patients undergoing PCI. Our main findings were as follows: (1) CYP2C19^*^2 and ^*^3 variants influence RPA, MAR and levels of sE-selectin, sCD40L, sP-selectin, MMP-9, and sVCAM-1 in patients following PCI. The efficacy of clopidogrel is reduced in IM+PM, compared to EM patients. (2) CYP2C19 EM patients have lower risk of MACE, compared with IM, PM, and IM+PM groups. (3) CYP2C19^*^2 and ^*^3 variants are independent risk factors of MACE in patients undergoing PCI.

Several studies have consistently reported that the CYP2C19^*^2 polymorphism is associated with lower antiplatelet response and increases the rate of recurrent cardiovascular events in Caucasian, Korean, and Chinese patients (Brandt et al., 2007; Shuldiner et al., 2009; Hochholzer et al., 2010; Oh et al., 2012; Liu et al., 2013). Moreover, the *CYP2C19* ^*^3 variant is associated with clopidogrel-mediated antiplatelet effects, especially in Asia. Since the incidence of the *CYP2C19* ^*^3 variant A allele is less than 1% in Caucasian populations, no carriers of *CYP2C19* ^*^3 were identified among 187 Caucasian healthy subjects and 237 Caucasian patients undergoing PCI in an earlier study (Geisler et al., 2008; Shuldiner et al., 2009). Conversely, the frequency of the *CYP2C19* ^*^3 variant in several Asian population studies was significantly higher at >20% (Lee et al., 2009; Yamamoto et al., 2011). Interestingly, CYP2C19^*^17, another functional variant, is additionally reported to contribute to the antiplatelet effect of clopidogrel (Sibbing et al., 2010), but its frequency is relatively rare (Chen et al., 2008; Sibbing et al., 2010). In the current study, we defined EM, IM, and PM patients treated with clopidogrel according to CYP2C19^*^2 and ^*^3 variant status to reflect CYP2C19 enzymatic activity more comprehensively and accurately.

To date, no large-scale clinical studies on clopidogrel resistance have been conducted, mainly due to the lack of clear and accepted definitions. Over several decades, the ADP-induced optical turbidimetric platelet aggregation assay, the traditional “gold standard” method to detect platelet function, has been employed to evaluate the antiplatelet efficacy of clopidogrel. However, in studies using this platelet function test, cutoff values for high on-treatment platelet reactivity established via receiver operating characteristic curve analysis were variable (Palmerini et al., 2014). A number of studies used other measures, such as SYNTAX score or platelet reactivity index, to investigate associations with risk of mortality, myocardial infarction and ST in patients with non–ST segment elevation acute coronary syndrome undergoing PCI (Palmerini et al., 2011, 2014). Clopidogrel blocks the platelet ADP receptor to inhibit platelet aggregation, in turn reducing expression of sCD40L and P-selectin and releasing them from platelets to prevent further platelet activation (Blake and Ridker, 2001; Steinhubl et al., 2002; Conde and Kleiman, 2003; Azar et al., 2006). Since the levels of sCD40L and sP-selectin are significantly different between the resting and activated states of platelets, they could be effectively evaluated as markers of platelet activation. These indexes can also be used for early assessment of the extent of platelet activation and anti-platelet effects to avoid the occurrence of ischemic and thrombotic events. We not only determined MAR and RPA, but also sCD40L and P-selectin levels in patients undergoing PCI after 24 h. Levels of these molecules were significantly lower in EM than IM+PM patients, supporting the conclusion that CYP2C19 metabolizers influence the anti-platelet effect of clopidogrel.

ST and in-stent restenosis involve platelet activation, inflammation and endothelial injury factor interactions. Clopidogrel has unique anti-inflammatory effects and could improve endothelial function (Ferroni et al., 2003; Heitzer et al., 2006; Husted et al., 2010; Zhang et al., 2015). Accordingly, we measured the levels of inflammation and endothelial injury factors, such as MMP-9, sVCAM-1, and sE-selectin, in patients undergoing PCI. Our results disclosed differences in MMP-9, sVCAM-1 and sE-selectin contents among *CYP2C19* variant groups. Specifically, MMP-9, sVCAM-1, and sE-selectin levels were significantly higher in CYP2C19 IM and PM than EM patients, implying that the CYP2C19 variants influence the metabolic activity of clopidogrel, further affecting its pharmacological ability to suppress inflammation and improve endothelial function.

We followed up 559 patients undergoing PCI treated with clopidogrel for 1 year, among which 519 were finally enrolled. Our data revealed a higher percentage of MACE in CYP2C19 IM and PM than EM patients. Moreover, the risk of MACE in IM+PM patients was 1.267 times higher than that in EM patients. Univariate analysis consistently showed that CYP2C19 metabolizers influence the occurrence of MACE after PCI. After adjustment for age, BMI, CHD risk factors (hypertension, diabetes, smoking), vascular lesions ≥3, frame length, bracket number and levels of platelet activity indicators (RPA, MAR, sCD40L, P-selectin, MMP-9, sVCAM-1, and sE-selectin), data from multivariate analysis showed that the risk of MACE in IM+PM patients is 2.664 times higher than that in EM patients. Our results are consistent with earlier studies showing that *CYP2C19* variants influence the risk of MACE in ACS or PCI patients treated with clopidogrel (Sibbing et al., 2009; Mega et al., 2010a; Wallentin et al., 2010; Jang et al., 2012). In both univariate and multivariate analyses, we did not establish platelet activity indicators or other inflammation and endothelial injury factors related to the risk of MACE. Previous studies have investigated sVCAM-1, E-selectin and other factors that influence the prognosis of acute myocardial infarction patients. The majority of subjects in our study were unstable angina patients undergoing PCI. Since these factors were examined after 24 h of PCI, the detected levels may not reflect occurrence of MACE, and further research is therefore required to establish this issue. Moreover, during the follow-up period, our study was limited to MACE data and did not include additional factors that could have influenced survival-free analysis, such as liver or kidney dysfunction.

Taken together, our results suggest that CYP2C19^*^2 and ^*^3 variants influence the levels of RPA, MAR, sE-selectin, sCD40L, sP-selectin, MMP-9, and sVCAM-1 in coronary heart patients undergoing PCI and modulate the drug efficacy of clopidogrel, further increasing the risk of MACE. Based on these findings, we propose that *CYP2C19* pharmacogenetic profiling may be beneficial for coronary heart patients undergoing PCI to predict whether treatment with clopidogrel is a feasible option. IM and PM patients may benefit from higher clopidigrel doses to improve efficacy and reduce the incidence of MACE.

## Author contributions

HS, JQ, and HC conceived and designed the experiments. HS, ZC, and HZ performed the experiments. JQ, QQ, and HS analyzed the data. HC and JQ contributed reagents/materials/analysis tools. HS, JQ, ST, and HC wrote the paper. All authors reviewed the final version of the manuscript.

## Funding

This work was supported by the National Natural Science Foundation of China (No. 81373838, No. 81403021, and No. 81273647) and Health and Family Planning Commission of Fujian Province Medical Innovation project (No. 2014-CX-5); the National Scientific foundation of China (No. 81503166, 81603208) and the Youth Foundation of Xiangtan Hospital in Central South University (2014Q08).

### Conflict of interest statement

The authors declare that the research was conducted in the absence of any commercial or financial relationships that could be construed as a potential conflict of interest.
